# Literature review of the research on nursing students’ professional self-concept

**DOI:** 10.1080/10872981.2022.2153396

**Published:** 2022-11-29

**Authors:** Yun Xu, Yongqi Liang, Hui Ye, Yue Xu

**Affiliations:** a Institute of Education, Nanjing University, Nanjing, Jiangsu Province, China; b School of Nursing, Nanjing University of Chinese Medicine, Nanjing, Jiangsu Province, China

**Keywords:** Nursing students, professional self-concept, theoretical connotation, measurement tools, influencing factors, effects, intervention experiments, literature review

## Abstract

**Objective:**

To understand the current situation and progress of nursing students’ professional self-concept, this review aimed to perform a general analysis of research related to the connotation of professional self-concept, measurement tools, influencing factors, effects, and intervention experiments.

**Methods:**

Three databases (Web of Science, PubMed, and CNKI) were searched for relevant articles. Research articles that met specific criteria were included, with identified articles initially screened by title and keyword. Then the abstracts were screened for relevance, and the full text was read for validation before inclusion. Descriptive analysis was performed with relevant findings from data retrieved from various sources.

**Results:**

Finally, 54 articles that met the criteria were included, which organised the connotation of self-concept of nursing speciality, and introduced six measurement scales, such as Professional Self-Concept of Nurses Instrument and Nurse’s Self-Concept Questionnaire. A total of 16 investigations on influencing factors were described, and the results showed that there were internal individual and external environmental factors. The professional self-concept was formed by analysing both factors. This paper described 17 effect surveys and found that professional self-concept had an important impact on students’ mental health, academic performance, and professional values, and so on. Eight intervention experiments including attribution training and hierarchical teaching were evaluated.

**Conclusions:**

Research articles on the professional self-concept included in this review were rich. These articles clarified the basic connotation of the concept, developed relatively mature measurement tools, found many influencing factors and effects, and proposed effective intervention strategies. They were of great value for understanding the professional self-concept and could provide a reference for scholars to conduct relevant research and practice. It also presents research prospects in this field, aiming to inspire future research.

## Introduction

The World Health Organization designated 2020 as the ‘International Year of the Nurse and the Midwife’ to recognise the important contributions made by these practitioners to global health. Nurses, as an important workforce in health services, have received increasing attention. As future nurses, nursing students’ professional self-understanding will have a crucial impact on their practice [1] and is significant in improving the quality of care and ensuring patient safety. ‘Professional self-concept’, as an important indicator of the ability to perceive oneself in a specific occupational environment, is a sensitive factor for predicting professional identity, job satisfaction, and career achievement [2], and has received extensive attention from nursing educators. This paper reviewed the relevant literature from the aspects of theoretical connotation, measurement tools, influencing factors, effects, and intervention strategies to understand the status and progress of the research field and provide a reference for future theoretical research and intervention practices.

## Materials and methods

This review includes a systematic search, research review, and descriptive analysis of existing literature.

### Search strategy

In September 2021, three databases, Web of Science, PubMed, and Child National Knowledge Infrastructure (CNKI), were searched using ‘professional self-concept of nursing student’ or ‘nursing students’ professional self-concept’ as key terms.

### Inclusion and exclusion criteria

The following inclusion criteria were used: describing nursing students’ professional self-concept theory, measurement tools, influencing factors, effects, and intervention experiments. The following exclusion criteria were used: unrelated to the subject matter, the same or similar to previous studies, and omitted data.

### Screening process

**Figure 1.**
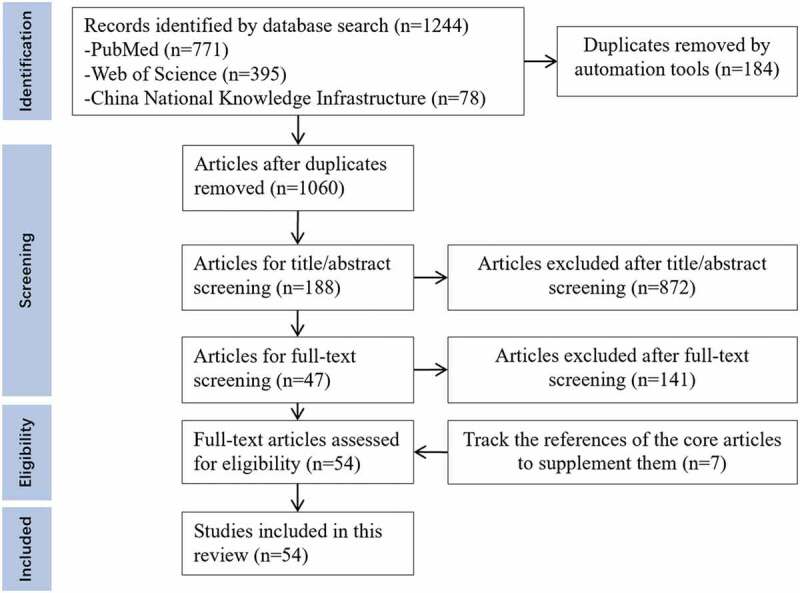
Literature retrieval and screening process.

A total of 1244 papers were obtained using the aforementioned search method, and duplicate papers were deleted. The authors screened the remaining papers to evaluate their relevance. Only those related to the research purpose and that met the inclusion criteria were included. We screened the abstracts of articles with relevant titles, and if deemed applicable, the full text was retrieved and reviewed. A total of 47 articles were selected for further analysis. Considering the continuity of theoretical development, we screened the list of references and searched the important literature on ‘self-concept’ and ‘professional self-concept’ manually. Based on this, seven additional articles were selected. Thus, 54 articles were included (Figure 1). The first author implemented the selection process, and the second reviewed and verified it.

### Charting the data

Microsoft Excel was used to create a data chart describing the literature on the measurement tools. Data were extracted independently from the articles and classified according to the following headings: title, author, compilation time, initial sample, scoring method, dimension (entry type), and internal consistency. A data chart describing the literature on the influencing factors was also created. Data were extracted independently from the articles and classified according to the following headings: author, title, publication year, research country, research type, number of participants, data collection time, and main research findings. To understand the quality of the intervention research in detail, the bias risk assessment and internal authenticity evaluation of the literature included in the intervention experiment were conducted using the risk of bias (RoB) 2.0 [3] and the JBI Evidence-based Centre evaluation manual in Australia [4].

### Collating, summarizing and reporting the results

We conducted a general analysis of the research on nursing students’ professional self-concept by combining all the relevant findings from the data retrieved from various sources, and systematically describing the concept connotation, measurement tools, influencing factors, effects, intervention means, and so on to evaluate the existing research status and confirm the knowledge gap, which can provide a reference for future research.

## Results

### Research on theoretical connotation

The idea of ‘self-concept’ was proposed in the continuous enrichment of the understanding of the ‘self’, and subcategories such as ‘academic self-concept’, ‘social self-concept’, and ‘professional self-concept’ were subsequently developed [5]. According to the symbolic interaction theory, the characteristics of nursing specialities were combined to propose the ‘nursing professional self-concept’ [6]. Therefore, to understand the professional self-concept of nursing students, the connotations of ‘self-concept’, ‘professional self-concept’ and ‘nursing professional self-concept’ were comprehensively clarified.

Connotation of self-concept

Self-concept refers to the relatively persistent self-experience and perception of an individual’s self-existence, a coping style appropriate to their environment [7], and a product of social experience established in interpersonal communication [8]. Through personal experience, self-reflection, and feedback from others, the consciousness and understanding of one’s experience and practice are gradually deepened [9], which comprises beliefs, attitudes, emotions, and values. Self-concept has two characteristics: 1) It is a multi-dimensional and multi-structural theoretical model [5].2) It is a dynamic development process subject to the constraints of age and cognitive development level, and incorporates unique habits, thoughts, abilities, and opinions to evaluate persistent beliefs and emotions. The functions of self-concept include self-guidance, self-expectation, self-explanation, and self-attribution of success or failure [10]. Active self-concept is a reflection of personality integrity, which is conducive to the realisation of other expected goals.

Connotation of professional self-concept

Professional self-concept is a subordinate concept of a specific component of self-concept that has a significant impact on behavioural achievements in professional fields and can predict individual professional achievements. It is a subjective process wherein individuals understand, apply, and enrich their self-concept for professional development. It is an attitude, morality, ideal, sense of responsibility, values, and so on toward the individual’s profession, which is generated based on gradual understanding [11]. In his career development theory, American psychologist Donald E. Super stated that self-knowledge and self-understanding of a career are core ideas of individual career development. According to career development theory, age groups 15 to 25 years is the ‘exploratory period’ and 18 to 21 years is the ‘transitional period’, a realistic adjustment of career expectations from ideal to reality. In the latter stage, professional self-concept is specifically critical for future career development and hence, that of students has attracted much attention.

Connotation of professional self-concept

The cultivation of students’ strong positive self-concept has been highly valued since nursing students’ self-esteem, self-confidence, and their influence on career choices received attention [12]. The professional self-concept of nurses was proposed. It is a profession-related and persistent perception of several professional self-attitudes during the transition from students to professional nurses. It reflects their professional self-understanding, self-esteem, and behaviour orientation, including knowledge, skills, flexibility, leadership, communication ability, and satisfaction [5]. Professional self-concept refers to the application of nurses’ professional self-concept in nursing, including views on the self, perception of one’s ability, cognition, understanding, and affection for the role. It is the integration of various ideas, principles, perceptions, and expectations of nurses, and has an important impact on nursing practice and related clinical work [13–15].

### Research on measuring tools

The proposal of self-concept is conducive to better grasping individuals’ self-understanding from social, psychological, and environmental perspectives. However, a high degree of abstraction of concepts impacts specific applications, and corresponding measurement tools need to be developed to facilitate the feasible application of concepts. Tennessee Self Concept Scale (TSCS), Wallance Self-Concept Scale (WSCS), Self-Description Questionnaires (SDQ), Multidimensional Self Concept Scale (MSCS), and other tools were subsequently introduced. The development of these scales also enabled nursing students to develop a measurement of their professional self-concept.

Professional self-concept of nurses instrument

David Arthur of Hong Kong Polytechnic University developed the Professional Self-Concept of Nurses Instrument in 1995. The scale included nursing diploma students in New South Wales, Australia, as a sample. Upon interviewing 24 graduating students and consulting nursing teachers and experts, a multi-dimensional structural model was established, and a questionnaire with seven dimensions and 56 items was initially completed. To test the questionnaire, 170 junior nursing diploma students in that region were selected as survey participants. Through structural analysis, factor analysis, and reliability evaluation, a formal scale comprising 27 items, including ‘practical skills (with flexibility, skills, and leadership dimensions)’, ‘satisfaction’, and ‘communication’, was developed, with a contribution rate of variance of 40.5% [16]. The scale was then used to investigate professional self-concept among registered nurses and nursing students in Canada and South Korea, and was applied to a multi-centre study of 1957 registered nurses from 11 countries and regions. The results of the multicentre survey further tested the effectiveness and stability of the tool. However, the differences in nursing cultures reflected in different countries and regions also guide future research [17–19].

Nurse’s self-concept questionnaire

Based on the multi-dimensional and multi-structural theoretical model proposed by Shavelson et al., Leanne Cowin, from Western Sydney University, developed Nurse’s Self-concept Questionnaire (NSCQ) in 2001. In this study, six-month informal interviews were conducted with clinical nurses in Sydney, Australia. Entries were extracted by encoding the qualitative interview data, and then reviewed and revised by an expert panel comprising six nursing experts and three self-concept experts. Initially, a preliminary questionnaire with 80 items was formed. It was used to investigate clinical registered nurses and nursing graduate interns from six major nursing colleges in Sydney. A total of 2118 individuals participated in the survey. Exploratory and confirmatory factor analyses were performed on the results, and a formal questionnaire with 36 items in six dimensions (comprehensive self-concept, colleague relationship, knowledge, care, communication, and leadership) was developed, with a variance contribution rate of 72.9% [13]. Hosseini revised the scale as assessed by a panel of experts for use in relevant studies of Iranian nurses [20].

Nurses’ self-concept instrument

Elizabeth Angel of Western Sydney University focused on the possible differences caused by cultural diversity in nurses’ professional self-concept among international students, specifically those from highly collectivist countries and local students. Referring to the NSCQ scale, she developed the Nurses’ Self-Concept Instrument (NSCI). This study selected 218 local and 35 international nursing students from an Australian university. The scale contains 14 items across four dimensions: care, knowledge, colleague relationships, and leadership. It could better clarify specific differences in the self-concept of specific cultural groups regarding cross-cultural analysis [15].

Other Relevant Measurement Tools

Moreover, according to the core connotation of nursing professional self-concept, tools such as Nurse’s Self-Description Form (NSDF) and the Porter Nursing Image Scale (PNIS) can also provide reference and enlightenment for us to measure nursing professional self-concept [21,22] (Table 1).

**Table 1.** Summary analysis of measuring tools.

Name of the tools	Initial sample	Author	Compilation time	Scoring method	Dimensions (number of entries)	Internal consistency
Professional Self-Concept of Nurses Instrument (PSCNI)	Australian nursing students	Arthur D.	1995	Likert4	Practical skills [including flexibility (6), skills (5), leadership (5)], communication (4), satisfaction (7)	0.59–0.85
Nurse’s Self-concept Questionnaire (NSCQ)	Australian nursing students	Cowin L	2001	Likert8	Comprehensive self-concept (6), colleague relationship (6), knowledge (6), care (6), communication (6), leadership (6)	0.83–0.93
Nurse’s Self-concept Questionnaire (Revised)	Iranian nurses	Hosseini A	2020	Likert5	Comprehensive self-concept (3), care (4), knowledge (5), colleague relations (3), communication (4), leadership (5)	0.83
Nurses’ Self-concept Instrument (NSCI)	Australian local and international nursing students	Angel E	2012	Likert8	Care (3), knowledge (4), colleague relations (3), leadership (4)	0.78–0.97
Nurse’s Self-description Form (NSDF)	American nurses	Dagenais F	1982	Likert7	Specialization (11), empathic ability (4), work ethics (4)	0.80–0.92
The Porter Nursing Image Scale (PNIS)	Colombian nurses	Porter R.T.	1991	Likert7	Interpersonal relationship (10), interpersonal rights (13), introspection ability (7)	0.57–0.88

### Research on influencing factors

The development of measurement tools has improved the feasibility of follow-up research on this concept. Hence, an empirical investigation of the professional self-concept of nursing students has been widely conducted. While measuring the status, many influencing factors were revealed.

Individual factors

Regarding individual factors, Yoo (2019) found that interpersonal relationships, positive psychological capital, religious beliefs, and learning performance affect the professional self-concept of nursing students [23]. CoPlu et al. (2018) found that female students had higher professional self-concept [24], and Yang (2020) found that critical thinking tendency, self-esteem, and creative integration ability positively impacted the professional self-concept [25]. A survey by Huang Miao et al. (2021) showed that professional identity, commitment, and affection were important factors that affected the professional self-concept [26]. Zhang et al. (2019) found that psychological distress and self-efficacy had significant effects on the professional self-concept [27]. Moreover, cultural background, prospective adaptation, and educational level also significantly affect the professional self-concept [15,28,29].

Environmental factors

Regarding environmental factors, Chang (2021) showed that the cultivation of critical thinking, a sense of belonging, and campus culture could continuously promote the development of nursing students’ professional self-concept [30]. Fangfei (2011) found that the level of social support positively affected professional self-concept [31]. Hae (2012) found that clinical satisfaction has a positive predictive effect [32]. Ou et al. (2016) found that the professional education environment had a significant positive correlation with the professional self-concept [33]. Fang et al. (2016) found that nurse image, development prospects, and labour rewards had a continuous impact [34]. Huang (2018) found that economic level and the reason for choosing majors also had significant effects [35]. Kim et al. (2020) found that the practical impact in the first clinical practice had a negative impact and the teacher-student relationship had an intermediary effect between the two [36]. Yi et al. (2020) also found that academic practice partnerships can facilitate professional self-concept among nursing students [37] (Table 2).

**Table 2.** Analysis of influencing factors.

Category	Source literature	Research objects	Sample size	Research type	Discovery factors
Individual factors	Yoo J H, 2019	Korean nursing students	355	Narrative research	Interpersonal relationship, positive psychological capital, religion, performance
	Coplu M., 2019	Turkish nursing students	619	Cross-sectional study	Gender
	Yang H.J.,2020	Korean nursing students	326	Cross-sectional study	Critical thinking tendency, self-esteem, creative integration ability
	Huang M, 2021	Chinese nursing students	207	Cross-sectional study	Professional identity, professional commitment, and professional affection
	Zhang W, 2016	Chinese nursing students	267	Cross-sectional study	Prospective adaptation
	Ge W.J, 2020	Chinese nursing students	304	Cross-sectional study	Educational level
	Zhang X.T, 2019	Chinese nursing students	262	Cross-sectional study	Psychological distress, self-efficacy
Environmental factors	Chang YC, 2021	Nursing students in Taiwan Province, China	395	Longitudinal study	Cultivation of critical thinking, sense of belonging, campus culture
	Angel E, 2012	Australian Local and International Nursing Students	253	Cross-sectional study	National cultural background
	Lv F.F, 2011	Chinese nursing students	185	Cross-sectional study	Social support
	Hae S.M.,2013	Korean nursing students	365	Cross-sectional study	Clinical satisfaction
	Huang F, 2016	Chinese nursing students	104	Longitudinal study	Nurse image, development prospect, labour remuneration
	Ou L.C, 2016	Chinese nursing students	527	Cross-sectional study	Professional education environment
	Huang H.M.,2018	Korean nursing students	132	Cross-sectional study	Economic level, reasons for choosing a major, pre-school results
	Kim.J.S.2020	Korean nursing students	184	Cross-sectional study	Practical impact of the first clinical practice
	Yi Y.J.,2020	Korean nursing students	243	Cross-sectional study	Academic practice partnership

### Effect research

In the empirical study, nursing students’ professional self-concept was studied as ‘result variable’, ‘dependent variable’, and ‘intermediary variable’ to explore the related effects and understand the impact on student development.

Influential effect

Regarding student psychology, Ge et al. (2020) surveyed 304 nursing students of different educational backgrounds in China and found that the definition of self-concept could positively affect students’ empathy [29]. Zhang et al. (2020) also found that professional self-concept positively impacted nursing undergraduates’ insight into their future [38]. Wang (2019) found that a positive professional self-concept could reduce the academic burnout level through a survey of 1083 nursing college students in China [39]. A survey of 137 nursing students in South Korea by Yang (2019) showed that professional self-concept had positively impacted students’ professional satisfaction and academic self-efficacy played an intermediary role [40]. Lv et al. (2011) found that professional self-concept impacted the employment pressure of nursing interns and that coping with stress could partly play an intermediary effect. The researchers also found that professional self-concept affected the emotion, intelligence, and depression of nursing students [41]. Hensel (2011) reported negative findings regarding nursing students in the USA and found that taking wellness courses improved the professional self-concept, but it failed to help students cope with stress when transitioning to professional roles [42].

Regarding student learning, Jahanbin et al. (2012) found in a study of 60 senior nursing students in Iran that the professional self-concept of students could significantly promote clinical performance [43]. Shin et al. (2012) conducted a cross-sectional survey of 1010 nursing students with different academic qualifications, and the results showed that professional self-concept and critical thinking significantly promoted clinical abilities [44]. Khalaila (2015) found that professional self-concept significantly impacted academic performance, while test anxiety and intrinsic motivation played an intermediary role. Moreover, achievement motivation has a remarkable regulatory effect [45]. Studies by Eskandari et al. (2019) and Nikolina et al. (2020) reported negative conclusions, and they investigated nursing students in Iran and Croatia, respectively. The results showed that professional self-concept did not correlate with students’ psychiatric nursing ability and clinical decision-making ability [46,47].

Regarding professional values, CoPlu et al. (2018) found that nursing students’ professional self-concept had a positive impact on professional values through a survey of 619 senior nursing students in Turkey [24]. Bi et al. (2021) completed a survey of Chinese master’s students of nursing majors and showed that professional self-concept was positively correlated with patients’ safety attitude, safety behaviour, and professional quality [48]. Lim et al. (2014) conducted a survey of 516 Korean nursing students and found that professional self-concept impacted students’ medical ethics consciousness [49]. A survey of 144 nursing students in South Korea by Lee et al. (2018) showed that it affected nursing students’ privacy protection behaviours toward patients [50].

Intermediary effect

Through a cross-sectional survey of 294 nursing students in South Korea, Hyun et al. (2018) found that professional self-concept played a role in increasing the impact of teacher-student interaction on job search anxiety, and there was a significant intermediary effect between the two [35]. Another cross-sectional survey conducted by Wu Han et al. (2021) on 122 nursing undergraduate interns in China demonstrated that professional self-concept played a partial intermediary role between learning involvement and humanistic care ability, and the intermediary effect accounted for 45.3% of the total effect [51]. The analysis of the current literature found that nursing students’ professional self-concept is rarely introduced into the study as an intermediary or regulatory variable, and they need to be further investigated.

### Intervention trial study

As professional self-concept plays an important role in student development, attention has been paid to cultivating nursing students’ positive professional self-concept, and experimental studies on related interventions have become increasingly abundant. The analysis of experimental results and evaluation of methodologies help provide evidence for future intervention practices.

Implementation of intervention experiments

Pu (2009) conducted a randomised controlled trial of 128 Chinese nursing interns and found that the experimental group using the reflective learning model had higher scores regarding professional self-concept [52]. Hensel (2011) measured 52 sophomores of nursing majors in the USA who participated in the wellness course before and after and found that participants had significant promoting effects on the leadership and communication dimensions of professional self-concept [42]. Ford (2015) used peer counselling to intervene with 43 American nursing undergraduates. A comparison of measurement results before and after the experiment showed that it could significantly improve the professional self-concept [53]. Seo et al. (2017) conducted a one-month intervention study on 59 Korean students in a two-year nursing program using a non-equivalent, pre-test/post-test control group design and found that post-mortem practice education could effectively improve the professional self-concept [54]. Jin (2019) found that narrative medicine teaching promoted students’ professional self-concept in a quasi-experimental study of 216 Chinese nursing interns [55]. Moreover, Wu (2014), Liang Fang-lian (2018), and Zhou Ye (2020) also conducted randomised controlled trials with Chinese students and found that attribution training, group cooperative learning methods, and hierarchical teaching modes effectively intervened in the professional self-concept [56–58].

Evaluation of research quality

**Figure 2.**
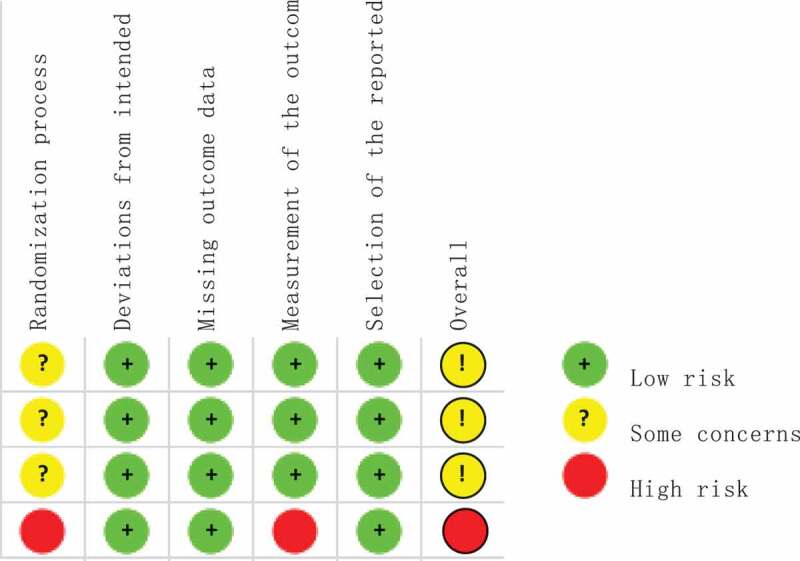
Summary of bias risk.

**Figure 3.**
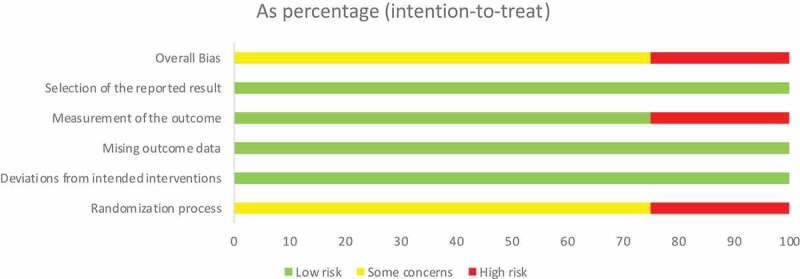
Proportions of items included in the study with risk of bias.

The RoB 2.0, bias risk assessment tool and evaluation manual of the Australian JBI Evidence-based Centre were used to evaluate the quality and authenticity of the aforementioned eight experimental research articles and assess the bias risk. Four randomised controlled trials (RCTs) [52,56–58] were used to assess the risk of bias using RoB 2.0. The results are shown in Figures 2 and 3 (Figures 2&3). Regarding the bias in the randomized process, two studies [52,58] did not report the random method of allocation sequence, and four did not report the allocation concealment, indicating that there was a possibility of selection bias. In the assessment of deviations from established interventions and outcome data deficiency, four studies showed good adherence to interventions and obtained outcome data from almost all subjects. However, these patients had a low risk of bias. In the bias assessment of the outcome measurement, the outcome indicator of one study [57] was a self-made scale, which was not tested for reliability and validity, and there was a certain risk of bias. The outcomes of the four studies were fully reported and the risk of bias was low. In summary, the descriptions of the random method and allocation concealment in the four articles were not sufficiently detailed, and there was a possibility of implementation and measurement bias. The four quasi-experimental studies [42,53–55] with a pre-test/post-test control group did not adopt the blinding or randomised grouping method. According to the evaluation manual issued by the JBI Evidence-based Centre, scales with good reliability and validity were used in all four studies as objective evaluation indices, and the outcomes of the subjects were fully reported. No samples were collected discretely during the study. The results were measured, and the data analysed. In conclusion, four quasi-experimental studies demonstrated good authenticity.

## Discussion

### Main findings

Existing research shows that the theory of professional self-concept for nursing students has been well developed under the overall framework of self-concept and its connotation, characteristics, and significance have been explained. Its multi-dimensional and multi-factor structural characteristics conform to the research orientation of psychometrics. Research on related measurement tools has rapidly developed. Hence, a solid foundation for research on the professional self-concept of nursing students was laid and related empirical research was conducted extensively. The influencing factors varied, namely, internal individual and external environmental factors. Under their combined effects, nursing students develop their professional self-concept. It exerts an important influence on students’ mental health, academic performance, and professional values, which is significant for their development. The experimental results prove that schools can effectively intervene in students’ professional self-concept through some explorations of reform; however, due to the limited quantity and quality of original studies, it is difficult to conduct an evidence-based evaluation. More evidence is required to support this conclusion. It can be seen that the professional self-concept is important and can be measured. Hence, it has attracted the attention of scholars. By combining existing research, we have also identified some aspects that need to be added.

### Cultural background of different countries

Cultural background and race may affect specific aspects of professional self-concept to promote or prevent professional behaviour [59]. A cross-border survey by Arthur showed significant differences in nursing self-concept and attributes among nurses from different cultures [19]. Research by Angel showed significant differences in the understanding of professional self-concept between native Australian and international students, specifically those from countries with high collectivist ideologies [15]. Cultural identity is an important factor in self-understanding [60]. Currently, a relatively clear theoretical development framework has been formed on ‘self-concept’, ‘professional self-concept’ and ‘nursing professional self-concept’, and relatively mature measurement tools have been developed. However, different nursing cultural backgrounds were not completely considered, and more attention should be paid to the heterogeneity of nursing cultures in different countries in future research.

### Influencing factors and mechanism

The development of self-concept is a dynamic process subject to the constraints of age and cognitive development. Nursing students’ professional self-concept was dynamic. It undergoes constant change with an in-depth study of courses and the impact of the internship [61]. It is also a longitudinal process of continuous understanding, adaptation, and internalisation. The development and change deserve attention. However, current research is mainly based on cross-sectional surveys, with relatively few longitudinal studies, which are not conducive to a more comprehensive understanding of the dynamic changes in the professional self-concept and to grasp the impact process and adopt targeted intervention methods more accurately. Moreover, the factors influencing professional self-concept vary. Nursing students formed their professional self-concept under the combined influence of internal and external factors, and the exploration of specific influencing mechanisms was important. However, such research is rare and needs to be further investigated.

### Quality of intervention evidence

Nursing education is necessary and can cultivate students’ strong professional self-concept [53]. It is important to cultivate a positive professional self-concept as it is conducive to maintaining a good physical and mental state, enhancing affirmation of self-ability, cultivating positivity and self-confidence in future clinical practice, and prompting the students to better participate in a nursing study to provide more effective care for patients [30]. Researchers have explored intervention measures and conducted effect evaluations through experimental research. From the quality evaluation of the experimental methodology, this kind of research had high adherence to interventions and completeness of results. Specifically, the overall quality of quasi-experimental research is high, while RCT research has a certain methodological bias. Future studies should further improve the standardisation of the research design and aim to improve the quality of evidence.

### Prospect of future research

Scientific research must be conducted using sound theoretical systems and profound theoretical accumulation. Future research on nursing students’ professional self-concept can be furthered in the following four aspects: First, strengthen the study and delve into existing research. Research on nursing students’ professional self-concept has a history of more than 30 years. The theoretical accumulation of research can be further enriched through in-depth research on previous literature, and that can be conducted more deeply based on communication with previous researchers. Second, the interpretation of concepts from different national and cultural backgrounds should be emphasised. Affected by multiple factors, such as medical level, health insurance strategy, and health service pricing, there are significant differences in nurses’ professional development levels, career development paths, and practice restrictions in different countries. It is necessary to understand students’ specific understanding from different cultural backgrounds and social environments. Third, we explored the influence mechanism. The professional self-concept of nursing students is systematically affected by a combination of internal and external factors. The significance of conducting only the confirmatory single-factor test is limited. Multiple factors should be considered when exploring the mechanism of this effect. Fourth, comprehensive and effective intervention strategies must be developed. Professional self-concept significantly impacts student development. Comprehensive and effective intervention strategies should be designed and experimentally verified. Hence, evidence-based evaluation can be conducted to provide more high-quality evidence and a reference for scientific intervention.

## Conclusions

Research articles on the professional self-concept included in this review were rich. These articles traced back to the development of research on professional self-concept of nursing students, clarified the basic connotation of the concept, developed relatively mature measurement tools, found many influencing factors and effects, and proposed effective intervention strategies. They were of great value for understanding the nursing students’ professional self-concept and could provide a reference for scholars to conduct relevant research and practice. At the same time, this review also proposes some areas for improvement in this research field, including ‘paying attention to different cultural backgrounds’, ‘exploring specific impact mechanisms’ and ‘improving the quality of evidence for intervention’. It also presents research prospects in this field, aiming to inspire future research.
